# Reflections on a Career in Research and Education

**DOI:** 10.1093/geroni/igaa057.3194

**Published:** 2020-12-16

**Authors:** Steven Zarit

**Affiliations:** Penn State University, State College, Pennsylvania, United States

## Abstract

This presentation will focus on three issues that were important in my career: family caregiving, international collaborations, and mentorship. Having spent considerable time studying family caregiving, I will highlight what I consider are fundamental issues that characterize family care and provide a necessary foundation for generating strong research questions. I will also suggest new directions for improving design and evaluation of interventions. International collaborations can help us broaden our understanding of aging and on care of older people. I had the good fortune to work with a great research team in Sweden. I will describe research we did on functioning and cognition among the oldest old. I also had the opportunity to see first-hand high-quality programs for older people that showed we can do better than accept mediocrity as the norm in care. Finally, I will discuss the importance of mentorship and bringing forward the next generation of researchers.

